# ﻿First insights into the phylogeny of the order *Cribrariales* (*Amoebozoa*, *Myxomycetes*), with the definite exclusion of the genus *Enteridium*

**DOI:** 10.3897/imafungus.16.159960

**Published:** 2025-10-02

**Authors:** Juan Carlos Zamora, Daniel Rodrigues, Iván García-Cunchillos, Carlos Lado

**Affiliations:** 1 Conservatoire et Jardin botaniques de Genève, Chemin de l'Impératrice 1, 1292 Chambésy, Switzerland Conservatoire et Jardin botaniques de Genève Genève Switzerland; 2 Department of Plant Sciences, University of Geneva, Quai Ernest-Ansermet 30, 1205 Genève, Switzerland University of Geneva Genève Switzerland; 3 Institute of Evolutionary Biology, Biological and Chemical Research Centre, Faculty of Biology, University of Warsaw, Warsaw, Poland Real Jardín Botánico, CSIC Madrid Spain; 4 Real Jardín Botánico, CSIC, Plaza de Murillo 2, 28014 Madrid, Spain University of Warsaw Warsaw Poland

**Keywords:** Bright-spored *Myxomycetes*, *
Cribraria
*, dictydine granules, *
Lindbladia
*, nomenclature, plasmodium, taxonomy, *
Trichiales
*

## Abstract

The order *Cribrariales* is among the least studied higher groups in the *Myxomycetes*, with numerous taxonomic problems and scarce molecular data available in public databases. Of the three genera currently accepted, viz. *Cribraria*, *Lindbladia*, and *Enteridium*, the last one shows a set of morphological characters clearly disagreeing with the two former ones. Using a representative sampling and two unlinked loci (nuclear and mitochondrial SSU), we assessed the phylogenetic relationships in the bright-spored *Myxomycetes (Lucisporomycetidae)* and concluded that the genus *Enteridium* must be excluded from the order *Cribrariales* and placed instead within the order *Trichiales*, family *Dianemataceae*. We provide detailed explanations of why this genus has been misclassified in previous studies, and define its morphological and molecular boundaries, performing two necessary new combinations. On the other hand, the phylogeny of the order *Cribrariales* s.str. shows three main lineages that are distinguished as three subgenera, viz. C. subg. Cribraria, C. subg. Dictydium, and C. subg. Ionokylix**subg. nov.**, the first one including the genus *Lindbladia* deeply nested and therefore treated as a heterotypic synonym of *Cribraria*.

## ﻿Introduction

A “natural” (phyletic) classification of the *Myxomycetes* has been aspired to for decades, and the combination of morphological and molecular data has allowed a more accurate and stable classification of the higher-level groups of these organisms. Early studies by Rostafiński (1874, 1875) and Lister (1894, 1925) roughly classified the *Myxomycetes* in two large groups, viz., *Lamprosporeae*/*Lamprosporales* and *Amaurosporeae*/*Amaurosporales*, according to the pigmentation of the spores, bright or dark, respectively. These two major lineages were retrieved by Fiore-Donno et al. (2010) in DNA-based phylogenies. At that time, the “bright-spored” clade comprised the orders *Trichiales* T. Macbr. and *Cribrariales* T. Macbr. s.l. (as *Liceales*), while the “dark-spored” clade included the orders *Physarales* T. Macbr., *Stemonitidales* T. Macbr. (as “*Stemonitales*”) s.l., and *Echinosteliales* G.W. Martin. Fiore-Donno et al. (2008) demonstrated the paraphyly of the order *Stemonitidales*, while Fiore-Donno et al. (2013) showed the paraphyly of both *Trichiales* and *Cribrariales* (as *Liceales*) in their original concepts. Leontyev et al. (2019) have proposed taxonomic amendments for all major groups of *Myxomycetes*, intending to construct a global classification exclusively based on monophyletic taxa, and providing new names in agreement with the International Code of Nomenclature for algae, fungi, and plants (ICN, Turland et al. 2025).

In its current concept, i.e., in strict sense, the order *Cribrariales*, with the sole family *Cribrariaceae* Corda, stands out as the earliest diverging lineage of the bright-spored clade (Fiore-Donno et al. 2013; Leontyev et al. 2019). Ramírez-Ortega et al. (2009) published a morphology-based phylogeny of the *Myxomycetes* with especial emphasis on *Cribraria* Pers., but their results substantially differ from *Myxomycetes* phylogenies based on molecular data and published around that time (e.g., Fiore-Donno et al. 2005, 2008, 2010) and later on. Very few *Cribrariaceae* sequences are available in public databases, possibly due to the difficulties for PCR amplification caused by high sequence divergence and the presence of long introns (Fiore-Donno et al. 2013). Remarkably, incorporating this information into the phylogenies revealed that the species *Lindbladia tubulina* Fr. is deeply nested within *Cribraria*, while *C. cancellata* (Batsch) Nann.-Bremek. represents an early-diverging lineage, sister to all other species of *Cribrariaceae* (Fiore-Donno et al. 2013). No other studies focused on the phylogenetic relationships of the *Cribrariaceae* have been published in the last decade and the taxonomic position of most of its members is far from being resolved.

Most current taxonomic treatments of the family *Cribrariaceae* include two genera, viz., *Lindbladia*, with the single accepted species *L. tubulina*, characterized by the aethaliate or pseudoaethaliate sporophores, usually with a more or less unperforated peridium (Hatano et al. 1996), and *Cribraria*, with ca. 50 sporocarpic species in which the remnants of the peridium persist in the form of a net, ribs, or nodes, and often also as a basal calyculus (Poulain et al. 2011; Lado and Eliasson 2022). Leontyev et al. (2019) included the genus *Enteridium* Ehrenb. (as “*Licaethalium* Rostaf.”) in the order *Cribrariales* s.str., based on morphological and molecular data from specimens identified as *E. olivaceum* Ehrenb. in a previous study (Leontyev et al. 2015). However, *E. olivaceum* lacks one of the diagnostic characters of the order *Cribrariales*, the dictydine granules, while it presents clustered spores, a feature previously unknown in the *Cribrariales*. These inconsistencies, coupled with the high divergence between the two DNA sequences ascribed to *E. olivaceum* in Leontyev et al. (2015) and their absence from the large analysis of Leontyev et al. (2019), advise for a revised study focused on the taxonomy and classification of this taxon.

Through the inference of a DNA-based phylogeny and detailed morphological studies of a representative taxon sampling, the present study aims at (i) reassessing the taxonomic placement of the genus *Enteridium*, establishing its boundaries with respect to the most closely related genera, (ii) providing an updated nomenclatural analysis of the names *Enteridium* and *Licaethalium* to contribute to their nomenclatural stability, and (iii) establishing the limits and diagnostic characters of the order *Cribrariales* s.str., revising the nomenclature at generic and infrageneric levels.

## ﻿Material and methods

### ﻿Sampling

49 new samples were selected for morphological and molecular study, comprising 31 specimens of *Cribrariales*, five of *Reticulariales*, one of *Liceales*, and 12 of *Trichiales*. This selection was complemented, for the molecular analyses, by a fair sampling of bright-spored *Myxomycetes* sequences analysed in Fiore-Donno et al. (2013) and García-Cunchillos et al. (2022), to retrieve the longest sequences of the targeted DNA regions (nrSSU and mtSSU, see below) while keeping most species and all major clades. In addition, we included the available genuine nrSSU sequences of *Cribraria* and *Enteridium* available in GenBank to compare them with our newly generated sequences, regardless of their length. This taxonomic sampling comprised a total of ca. 100 species and ca. 20 genera in the four recognized orders of bright-spored *Myxomycetes*. Finally, 18 specimens from the dark-spored clade (families *Didymiaceae* and *Physaraceae*) from García-Martín et al. (2023), with nearly complete nrSSU and mtSSU sequences, were selected as outgroup to root the phylogenies, based on Fiore-Donno et al. (2010). The list of newly sequenced specimens selected for phylogenetic analyses can be found in Table 1.

**Table 1.** Specimens from which Sanger DNA sequences were newly generated in this study, with geographical origin, voucher information and GenBank accession numbers.

Taxon	Country	Herbarium voucher (collector number)	Genbank asession numbers			
			nrSSU-1	nrSSU-2	nrSSU-3	mtSSU
*Cribraria argillacea*	Switzerland	G00586004 (D. Rodrigues 8/2022)	PV943897	PV943897	PV943897	PV943852
*Cribraria argillacea*	Sweden	G00586061 (D. Rodrigues 39/2023)	PV943898	PV943898	–	PV943853
*Cribraria aurantiaca*	Spain	G00586271 (D. Rodrigues 225/2023)	–	PV943899	PV943900	PV943854
Cribraria cancellata var. cancellata	Spain	G00586046 (D. Rodrigues 38/2022)	PV943901	PV943901	–	PV943855
Cribraria cf. cancellata var. fusca	Chile	MA-Fungi 80564 (Lado 17172)	PV943902	–	–	PV943856
Cribraria cancellata var. fusca	France	MA-Fungi 94033 (M. Meyer 49156)	PV943903	–	–	PV943857
*Cribraria confusa*	France	MA-Fungi 89818 (M. Meyer 33037)	PV943904	–	–	PV943858
*Cribraria cribrarioides*	Japan	MA-Fungi (Lado 25828)	–	–	–	PV943859
Cribraria cf. intricata	Sweden	G00586150 (D. Rodrigues 46/2023)	PV943905	PV943905	PV943905	PV943860
Cribraria aff. lepida	Australia	MA-Fungi (TVDH 531)	PV943906	–	–	PV943861
Cribraria cf. macrocarpa	Finland	G00586115 (D. Rodrigues 103/2023)	PV943907	PV943907	PV943907	PV943862
*Cribraria meylanii*	Norway	O F-371517 (E.W. Johannesen s/n)	PV943908	–	–	PV943863
*Cribraria meylanii*	Switzerland	G00586187 (M. Wilhelm s/n)	PV943909	PV943909	PV943909	PV943864
*Cribraria minutissima*	Australia	MA-Fungi (Lloyd, S.J. 2018)	–	–	–	PV943865
*Cribraria minutissima*	Spain	MA-Fungi (Lado 27818)	–	–	–	PV943866
*Cribraria mirabilis*	Norway	O F-371516 (K.A. Mandal s/n)	PV943910	–	–	PV943867
Cribraria cf. piriformis	Switzerland	G00586042 (D. Rodrigues 34/2022)	PV943911	PV943911	PV943911	PV943868
*Cribraria purpurea*	Finland	G00586358 (H. Koskinen JX.164888#7)	PV943912	PV943912	–	PV943869
*Cribraria rubiginosa*	The Netherlands	G00586350 (M. Meyer 30448)	–	–	–	PV943870
*Cribraria rufa*	Finland	G00586359 (H. Koskinen JX.1648888#5)	PV943913	PV943913	PV943913	PV943871
*Cribraria rufa*	Finland	G00586360 (H. Koskinen JX.1640936#3)	PV943914	PV943914	PV943914	PV943872
*Cribraria tubulina*	Spain	G00586002 (D. Rodrigues 33/2022)	PV943924	PV943924	PV943924	PV943879
*Cribraria tubulina*	Spain	G00586060 (D. Rodrigues 135/2023)	PV943925	PV943925	–	PV943880
*Cribraria violacea*	Switzerland	G00586003 (D. Rodrigues 9/2022)	–	–	PV943915	PV943873
*Cribraria vulgaris*	Spain	MA-Fungi 61604 (Oltra 7197)	PV943916	–	–	PV943874
*Cribraria zonatispora*	Spain	G00586255 (D. Rodrigues 208/2023)	–	–	–	PV943875
*Cribraria* sp.	Australia	MA-Fungi (Lloyd, S.J. 1988)	PV943917	PV943918	PV943919	PV943876
*Cribraria* sp.	Australia	MA-Fungi (TVDH 568)	PV943920	–	PV943921	PV943877
*Cribraria* sp.	Switzerland	G00586314 (J.C. Zamora s/n)	PV943922	PV943922	PV943923	PV943878
*Licea castanea*	Finland	G00586172 (D. Rodrigues 83/2023)	–	–	PV943926	PV943881
*Arcyria cinerea* s.l.	Switzerland	G00586054 (D. Rodrigues 13/2023)	PV943927	–	PV943928	PV943882
*Enteridium corticatum*	France	G00586351 (M. Meyer 24378)	–	–	–	PV943883
Enteridium cf. liceoides	France	G00586352 (B. Woerly 3435a)	PV943929	–	–	PV943884
Enteridium cf. liceoides	Spain	MA-Fungi 71211 (L.C. Rey CR77M99)	–	–	–	PV943885
*Enteridium olivaceum*	Spain	MA-Fungi 57408 (Oltra 4138)	PV943930	–	–	PV943886
*Enteridium olivaceum*	Spain	MA-Fungi 39466 (Oltra 1730)	PV943931	–	–	PV943887
*Enteridium olivaceum*	France	G00586353 (M. Meyer 47160)	PV943932	–	–	–
Enteridium cf. simulans	France	G00586354 (M. Meyer 29889)	–	–	–	PV943888
*Enteridium variabile*	Finland	G00586171 (D. Rodrigues 86/2023)	–	–	–	PV943889
*Enteridium variabile*	Finland	G00586173 (D. Rodrigues 123/2023)	PV943933	–	PV943934	PV943890
*Enteridium variabile*	Finland	G00586174 (D. Rodrigues 115/2023)	–	–	PV943935	PV943891
*Enteridium variabile*	Finland	G00586169 (D. Rodrigues 98/2023)	PV943936	–	PV943937	–
*Lycogala flavofuscum*	Italy	G00586355 (M. Meyer 22037)	–	PV943938	–	PV943892
*Lycogala flavofuscum*	Spain	G00586059 (D. Rodrigues 136/2023)	PV943939	PV943939	PV943939	PV943893
*Lycogala leopardinum*	Switzerland	G00586053 (D. Rodrigues 12/2023)	–	PV943940	–	PV943894
*Reticularia lycoperdon*	France	G00586356 (M. Meyer 22374)	PV943941	–	–	PV943895
*Tubifera ferruginosa*	France	G00586357 (M. Meyer 31696)	PV943942	PV943942	PV943942	PV943896

### ﻿Morphology

The morphological characters of the studied specimens were gathered from both fresh samples, collected in the field or raised in moist chamber cultures (Gilbert and Martin 1933), and from dried specimens of the herbaria G (including the collections of M. Meyer), MA-Fungi (including the collections from C. Lado, S. Lloyd, and T. Van Der Heul), and O (https://sweetgum.nybg.org/science/ih/), and the private collections of H. Koskinen (Finland), M. Wilhelm (Switzerland), and B. Woerly (France). Herbarium specimens were directly observed and photographed with a Leica M165C stereomicroscope, coupled with a DMC2900 digital camera, and mounted in Hoyer’s medium (Martin and Alexopoulos 1969) or occasionally in water for study and photography under a Leica DM2000 light microscope, coupled with a DMC5400 digital camera. Some representative specimens were selected for study under the scanning electron microscopes (SEM) of the Royal Botanic Garden of Madrid, by using a Hitachi S-3000N SEM and a Jeol T330A (Tokyo, Japan) at 10–15 kV. All samples were dehydrated in acetone series starting at 30% up to 100% for 15 minutes each step, dried with the critical point technique and coated with gold in a Balzers SCD 004 sputter coat. The specimens were determined by using the general identification keys (e.g., Lister 1925; Martin and Alexopoulos 1969; Nannenga-Bremekamp 1974, 1991, 2022; Neubert et al. 1993; Lado and Pando 1997; Poulain et al. 2011), specific taxonomic treatments (e.g., Nannenga-Bremekamp 1958, 1962, 1964, 1971; Hatano 1988; Keller et al. 1988; Hatano et al. 1996), as well as information directly retrieved from the protologues. Our observations were complemented with those made by other authors and available in the literature.

### ﻿DNA extraction, PCR amplification and sequencing

Two to ten adjacent sporocarps, or an equivalent amount for aethaliate species, were taken from each specimen for DNA extraction. Each sample was transferred to a 1.5 ml tube containing one tungsten bead, frozen at -20 °C for at least one hour, and then mechanically disrupted in a TissueLyser II. DNA extractions were made using two commercial kits: the Norgen Biotek Corp Plant/Fungi DNA Isolation Kit (Canada; Product # 26200) following the manufacturer protocol with two minor modifications: i) samples were incubated in the lysis buffer for at least one hour, and ii) DNA was eluted twice with 50 µl of the elution buffer each time, and the DNeasy Plant Mini Kit (Qiagen Product # 69106) with two minor modifications: i) samples were incubated in the lysis buffer overnight, and ii) DNA was eluted twice with 80 µl of the elution buffer each time. When samples were too small or scarce, the eluted DNA was concentrated by evaporation in a refrigerated vacuum centrifuge.

Two unlinked DNA regions were selected for this study: the nuclear small subunit ribosomal RNA or 18S RNA for being the classical region to reconstruct phylogenetic relationships in the *Myxomycetes*, and the mitochondrial small subunit ribosomal RNA or mtSSU, which has shown good resolution for internal nodes of the phylogeny in both dark and bright-spored *Myxomycetes* and it is comparatively easy to amplify (García-Cunchillos et al. 2022; Lado et al. 2022; García-Martín et al. 2023). Due to the length and presence of introns in the 18S RNA, we amplified and sequenced this region in three overlapping fragments: S1, S2 and S3 as defined by García-Cunchillos et al. (2022). To improve DNA amplification success, especially in *Cribrariales*, new primers were designed. The list of primers used in this study can be found in Table 2.

**Table 2.** Primers used in this study.

DNA region	Primer name	Sense	Sequence (3’–5’)	Taxonomic range	References
nrSSU-1	S1	F	AACCTGGTTGATCCTGCC	Bright-spored	Fiore-Donno et al. (2008)
	SR4Cri	R	CACCAGACTTTCCCACT	* Cribrariales *	This study
	SR4Lic	R	CCAGACTTGTCCTCCAAT	Bright-spored except *Cribrariales*	This study
	nSSU2Ret-F	F	AGAGGATTAGGGTTTGATCCT	* Reticulariales *	This study
					
nrSSU-2	nSSU2Cri-F	F	TTCYAAGGAWGGCAGCAG	Bright-spored except *Reticulariales*	This study
	SR12Cri	R	GACTACAACGGTATCTAATC	* Cribrariales *	This study
	SR12Tri_bis	R	GGACTACGATGGTATCTGAT	* Trichiales *	This study
	SR12Lic†	R	CTGGACTACTGTGGTATCTGA	*Reticulariales* and *Liceales*	Fiore-Donno et al. (2010)†
					
nrSSU-3	S5Bright	F	GGTGAAATCCGWTGAYCCT	Bright-spored	This study
	nSSUBright-34-F2	F	TGGTGCATGGYCGTTCKTA	Bright-spored	This study
	nuSSUBright-R1	R	GATCCTTCTGCAGGTTCACC	Bright-spored	This study
					
mtSSU	Kmit_F	F	AGTGTTATTCGTGATGACTGG	* Myxomycetes *	Lado et al. (2022)
	Kmit_R	R	CGAATTAAACCACATCTCCACC	* Myxomycetes *	Lado et al. (2022)

†Modified to approach melting temperatures of other primers.

Each PCR reaction contained 12.5 µl of Red *Taq* DNA Polymerase 2× Master Mix 1.5 mM MgCl_2_ (VWR®), 0.5 µl of each primer, forward and reverse, at 10 mM, 3–5 µl of template DNA, and completed with bidistillate water for a final volume of 25 µl. PCR conditions for the amplification of each region were: initial denaturation (94 °C, 1 min), 30 cycles of denaturation (94 °C, 30 sec), annealing (1 min at 52 °C for mtSSU, or 1 min at 55 °C for nrSSU, with minor adjustments for specific samples), and extension (72 °C, 3 min), and a final extension step (72 °C, 10 min). PCR amplifications were checked by electrophoresis in a 1–1.5% agarose gel and 1× TAE buffer. Successful amplifications were purified with ExoProStar (illustra, United Kingdom) and sent to sequence, in both directions and with the same primer pairs, at Macrogen Europe facilities. Sequences of nrSSU and mtSSU from G00586039 (C. cancellata var. fusca) and G00586052 (*C. vulgaris*) were extracted from low coverage genome sequencing data generated for an ongoing project (unpublished data).

### ﻿Bioinformatics

Newly generated sequences were edited with Sequencher® v5.4.6 (Gene Codes Corporation) and trimmed to exclude primer sites and poorly read positions. These sequences were aligned together with those retrieved from GenBank with PASTA v1.9 (Mirarab et al. 2015) using the following settings: positions present in only one or two of the sequences masked, MAFFT (L-INS-i) as the algorithm, OPAL as the merger, fasttree as the tree estimator, and 10 (mtSSU) or 20 (nrSSU) iterations, retaining the best likelihood scored alignment. The resulting alignments were minimally adjusted, mainly towards the ends of some incomplete sequences. Regions that were highly variable and ambiguously aligned were removed, retaining the most conserved blocks for subsequent analyses as suggested by Fiore-Donno et al. (2013) and García-Cunchillos et al. (2022). The final alignments are available in http://purl.org/phylo/treebase/phylows/study/TB2:S32191.

Phylogenies were estimated by Maximum Likelihood (ML) and Bayesian Inference (BI) approaches. ML analyses were performed in IQ-TREE v1.6.12 (Nguyen et al. 2015) for each of the two loci separately to check for incongruences across datasets. An incongruence was considered when significantly supported contradictory topologies were obtained from each dataset, using a threshold of 95% ultrafast bootstrap as indicative of significant clade support. No such incongruences were detected and then the datasets were combined for further analysis. ML analyses of the combined dataset were equally performed in IQ-TREE v1.6.12, preliminary partitioning the data into the mtSSU and the nrSSU partitions, the models and final partitioning scheme of which were estimated by the partition merging option and the integrated version of ModelFinder (Kalyaanamoorthy et al. 2017). Ten independent replicates were performed, retaining the tree with the best likelihood score. Branch support was assessed by standard bootstrap (Felsenstein 1985), performing 1000 replicates. Branch support was additionally evaluated by 1000 replicates of the Shimodaira-Hasegawa-like approximate likelihood ratio test (SH-like aLRT, Guindon et al. 2010), to account for very short branches supported by nearly no data but that could receive spurious support by bootstrap. Finally, we calculated the transfer bootstrap expectation (TBE, Lemoine et al. 2018) in the online platform of BOOSTER (https://booster.pasteur.fr/), using 1000 standard bootstrap replicates, to account for the effect of rogue taxa in branch supports. Threshold values for significant support were considered as BS ≥ 70%, SH-like aLRT ≥ 75%, and TBE ≥ 80%.

**Figure 1.**
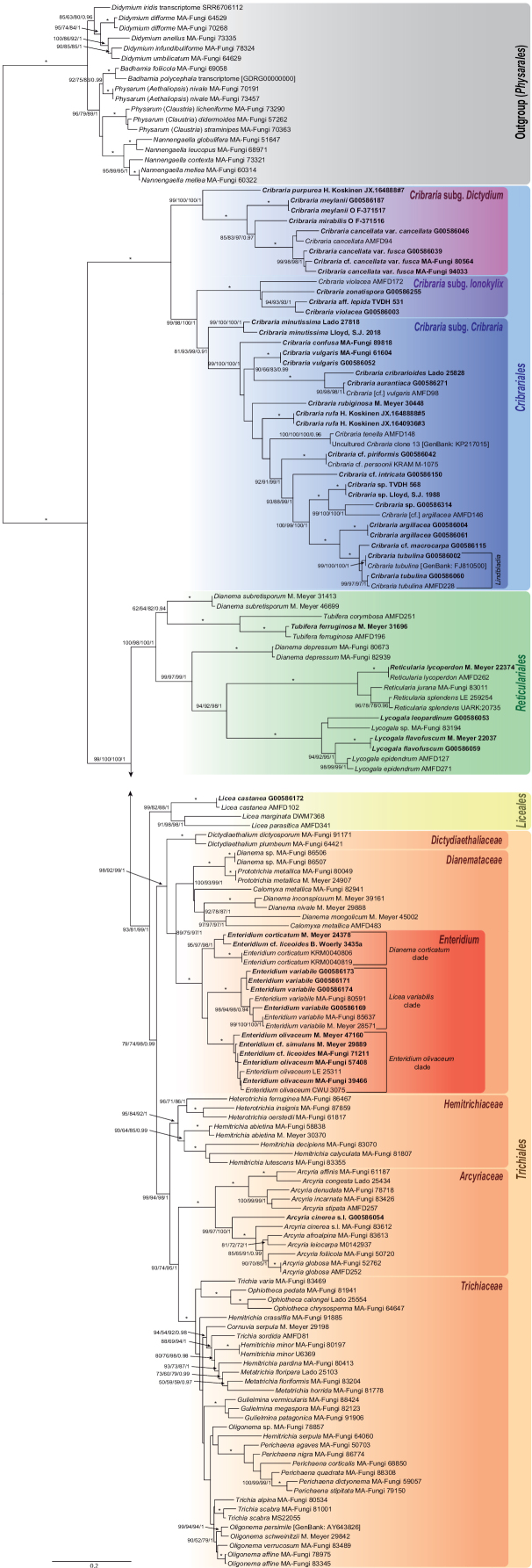
Maximum likelihood phylogenetic tree of the bright-spored *Myxomycetes (Lucisporomycetidae)* based on the concatenated dataset of nrSSU and mtSSU DNA sequences. Values on branches represent the SH-like aLRT, the standard bootstrap (BS), the transfer bootstrap expectation (TBE), and the posterior probabilities (PP). These values are only indicated when SH-like aLRT, BS, and TBE ≥ 50%, and PP ≥ 0.9. Maximum support (SH-like aLRT = 100%, BS = 100%, PP = 1, and TBE = 100%) is denoted by an asterisk. Samples with newly generated sequences are in bold. The scale bar indicates the average number of substitutions per site.

BI analyses were done in MrBayes 3.2.7a (Ronquist et al. 2012) with the same partitioning scheme used for the ML analysis and unlinking all parameters except topology. Models of evolution were estimated by model jumping (Huelsenbeck et al. 2004) with nst=mixed and allowing a proportion of invariant sites and a gamma-distributed rate heterogeneity across sites. We ran four simultaneous analyses with six chains each, five of them heated with a temperature factor of 0.1 after evaluating the swapping values among chains in a preliminary run. The prior on branch lengths was set as unconstrained:gammadir(1,0.07386,1,1) based on the best replicate of the ML analysis. The analyses were run for 1×10^7^ generations sampling every 1000^th^ tree. Parameters and trees were summarized after discarding the first half of the runs as burn-in, and a 50% majority rule consensus tree with average branch lengths and posterior probability (PP) values was computed from the remaining trees. Branches were considered significantly supported when PP ≥ 0.95. Trees were edited with FigTree v1.4 and the best replicate of the ML tree is shown in Fig. 1.

## ﻿Results

### ﻿Sporophore development in the family Cribrariaceae

**Figure 2.**
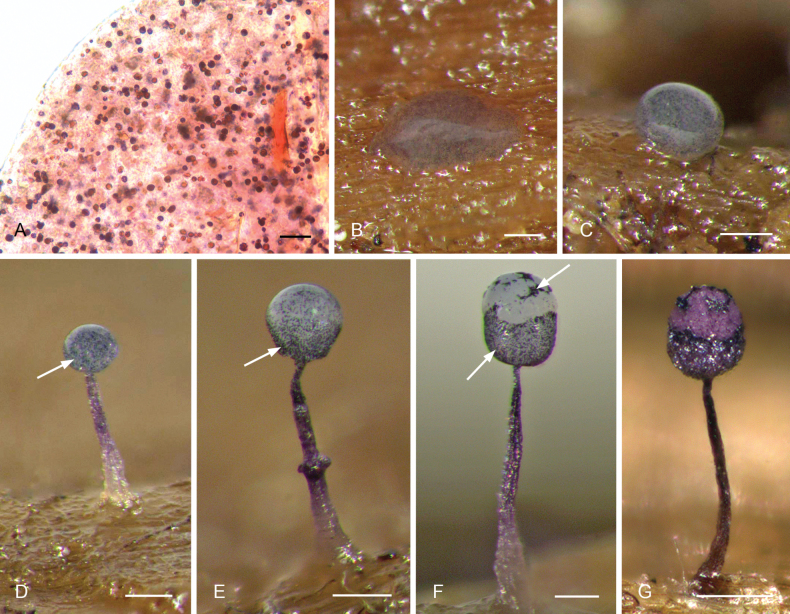
Sporophore development in *Cribrariales*, as observed in moist chamber culture of *Cribraria violacea* (G00586043). A Emerging plasmodium under the light microscope showing randomly distributed dictydine granules in the cytoplasm; B flat emerging plasmodium on the substrate; C round sporophore initial after concentration of the plasmodium; D stalked developing sporocarp still with an immature sporotheca with dictydine granules randomly distributed in the cytoplasm; E dictydine granules migrating towards the peridium; F sporocarp with fully formed peridium with numerous dictydine granules and pale immature spore mass practically without granules; G fully mature sporocarp with its final pigmentation. Arrows indicate the concentration of dictydine granules. Scale bars: 10 µm (A); 0.1 mm (B–G).

Our observations in moist chamber cultures (Fig. 2) show that the plasmodium in the *Cribrariaceae* is usually visible only when it is ready to fructify, remaining within the substrate before this state. This “emerging” or “mature” plasmodium is flat and already contains fully formed dictydine granules, randomly distributed within the cytoplasm (Fig. 2A, B). Superficial plasmodia then concentrate to form one or more rounded sporophore initials (Fig. 2C), retaining the same colour and distribution of dictydine granules. In species with sporophores in the form of stalked sporocarps, a subhypothallic stipe develops, sometimes retaining part of the dictydine granules, while the immature sporotheca still remains rounded and with dictydine granules randomly distributed (Fig. 2D). In a more advanced state of development of the sporophores, numerous dictydine granules (often the majority) migrate towards the surface and concentrate on the developing peridium (Fig. 2E). At this point, the immature spore mass clears up in many, but not all species, and may contrast with the young peridium (Fig. 2F). In a few species, notably in *C. cancellata*, *C. purpurea* Schrad. and allies, but also in *Lindbladia tubulina*, the immature spore mass never clears up, remaining dark-coloured until the maturation of the sporophores. Our observations indicate that many dictydine granules in those *Cribraria* species remain within the mature spore mass, possibly contributing to its dark colour, while in *L. tubulina* the intricate structure of the aethalium or pseudoaethalium contribute to its opacity, regardless of the position of the dictydine granules.

### ﻿Phylogeny and characterization of target clades

116 new sequences were generated in this study (Table 1). The final dataset contained 1742 characters of which 1164 were parsimony-informative and 76 were singleton sites. The evolutionary models selected by ModelFinder for the ML analysis were GTR+F+I+G4 for the mtSSU partition and SYM+I+G4 for the nrSSU partition. The overall topology of the bright-spored *Myxomycetes* tree (Fig. 1) shows four main imbricate and supported (aLRT = 79–100%, BS = 74–100%, TBE = 88–100%, PP = 0.99–1) monophyletic groups, which are distinguished as the following four orders: (i) *Cribrariales*, (ii) *Reticulariales*, (iii) *Liceales*, and (iv) *Trichiales*.

**Figure 3.**
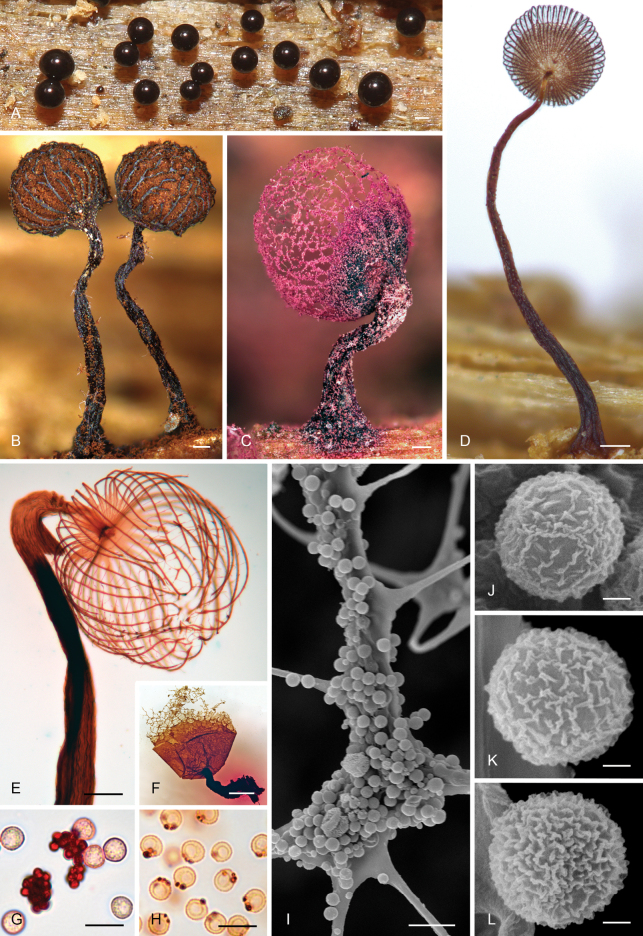
Morphological traits in Cribraria subg. Dictydium. A Blackish sporophore initials of C. cancellata var. fusca (G00586039); B–F sporocarpic sporophores of: *Cribraria mirabilis* (B, O F-371516), *C. purpurea* (C, Koskinen JX.1648888#7), C. cancellata var. fusca (D, G00586039), C. cancellata var. cancellata (E, G00586046), and *C. meylanii* (F, O F-371517), the last two mounted in Hoyer’s medium; note the pinkish-purplish pigments leaching from the stalks of these last two samples, more visible in image F; G spores and free clusters of dictydine granules in *C. purpurea* (Koskinen JX.1648888#7, in water); H spores with attached dictydine granules in *C. meylanii* (G00586187); I node of *C. purpurea* (MA-Fungi 89881) with abundant free dictydine granules; J–L spores under the scanning electron microscope of C. cancellata var. fusca (J, MA-Fungi 94033), C. cancellata var. cancellata (K, MA-Fungi 81827), and *C. purpurea* (L, MA-Fungi 89881). Scale bars: 0.1 mm (A–E); 0.5 mm (F); 10 µm (G–I); 1 µm (J–L).

The order *Cribrariales* s.str. includes all analysed species of *Cribraria* and *Lindbladia* (Fig. 1). Within it, we distinguish three main lineages. The earliest-diverging lineage (Cribraria subg. Dictydium, see discussion) is strongly supported in all analyses (SH-like aLRT = 99%, BS =100%, TBE = 100%, PP =1) and includes species of *Cribraria* (Fig. 3) with a very dark brown to blackish emerging plasmodium and sporophore initials (Fig. 3A), sometimes with purplish tinges, and with dictydine granules that are not only located at the peridium and stalk of mature sporophores, but also abundantly present in the spore mass, either as packages of granules (as in *C. purpurea*, Fig. 3G, I) or being regularly seen attached to some spores in microscopic preparations (*C. meylanii* Brândza, *C. mirabilis* [Rostaf.] Masee, and *C. cancellata* s.l., Fig. 3H). All species in this group form clearly stalked sporocarps and some produce pink or purplish pigments, often leaching when the sporocarps are mounted in Hoyer’s medium (Fig. 3E, F). The mature peridium may be ribbed, reticulated, or with a mixture of ribs in the lower part and a reticulate net in the upper part, with or without a basal calyculus (Fig. 3B–F). The spore ornamentation seems quite consistent, always in the form of low and irregular short ridges or short crests to elongated warts (Fig. 3J–L). No secondary ridge-like ornamentation or polyhedric spores have been observed.

**Figure 4.**
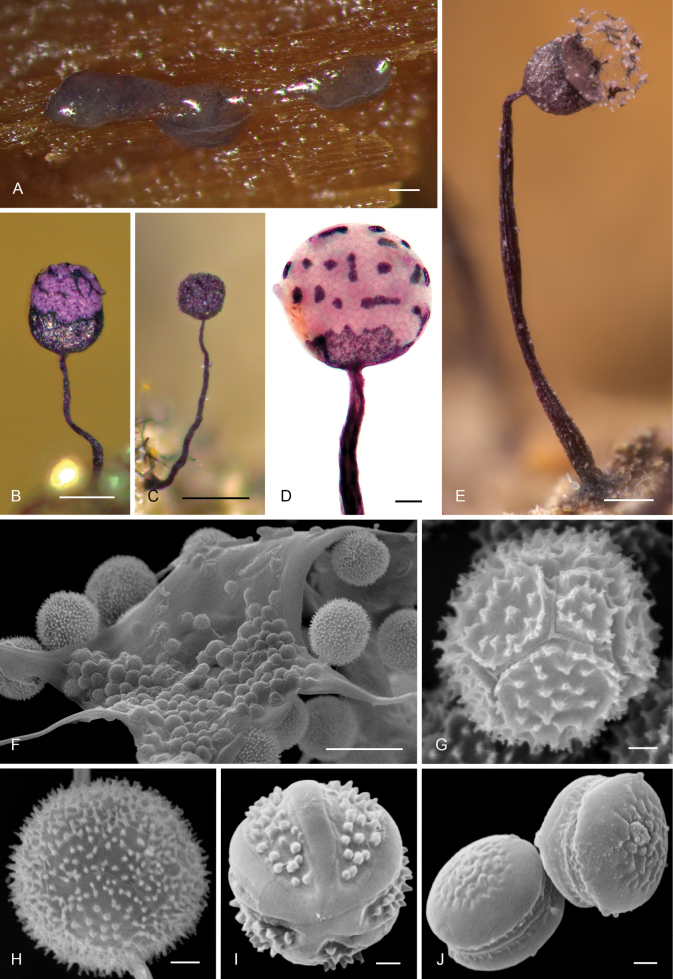
Morphological traits in Cribraria subg. Ionokylix (*C. fragilis* and *C. tecta* are included based on morphology only, see discussion). A Emerging violaceous plasmodia of *C. violacea* (G00586043); B–E sporocarpic sporophores of *C. violacea* (B, G00586003), *C. zonatispora* (C, D, G00586255), the one in image D not fully mature and mounted in Hoyer’s medium, and C. aff. lepida (E, TVDH 531); F inner side of a node of C. aff. lepida (TVDH 531) with encrusted dictydine granules; G–J spores under the scanning electron microscope of *C. tecta* (G, Hooff 8832, uncited isotype in MA-Fungi), C. aff. lepida (H, TVDH 531), *C. fragilis* (I, MA-Fungi 46919, isotype), and *C. zonatispora* (J, MA-Fungi 37500, isotype). Scale bars: 0.1 mm (A–C, E); 20 µm (D); 10 µm (F); 1 µm (G–J).

A second lineage of *Cribrariales* (Cribraria subg. Ionokylix, see discussion) received maximum support and comprises the species with distinctly violet to dark violet sporophores, always clearly stalked (Fig. 4). The emerging plasmodia in this group have violaceus colours (Fig. 4A), often dark violaceous or dark purplish to greyish violet. In mature sporophores, the dictydine granules are concentrated in the peridium (Fig. 4F) and sometimes in the stalks (scattered granules occasionally present in the spore mass). The peridium is always represented by a calyculus at the base of the sporotheca and usually by a more or less developed net with flat nodes at the upper part (Fig. 4B, E); the connecting threads or even the entire net are rarely absent or evanescent (e.g., *C. zonatispora* Lado, Mosquera and Beltrán-Tej., Fig. 4C, D). The spores in this group display a large morphological variation, and these ornamentations are often diagnostic for species-level identification, such as the spiny elements of *C. tecta* Hooff and C. aff. lepida Meyl. (Fig. 4G–I), the cingulate spores of *C. zonatispora* (Fig. 4J), or the spores with several raised smooth bands arranged in longitudinal arcs in *C. fragilis* Lado and Estrada (Fig. 4I), the last two being unique in the order. A secondary ornamentation formed by long ridges creating an ample reticulum with polyhedric meshes is present in some species (e.g., *C. violacea* Rex and *C. tecta*, Fig. 4G).

**Figure 5.**
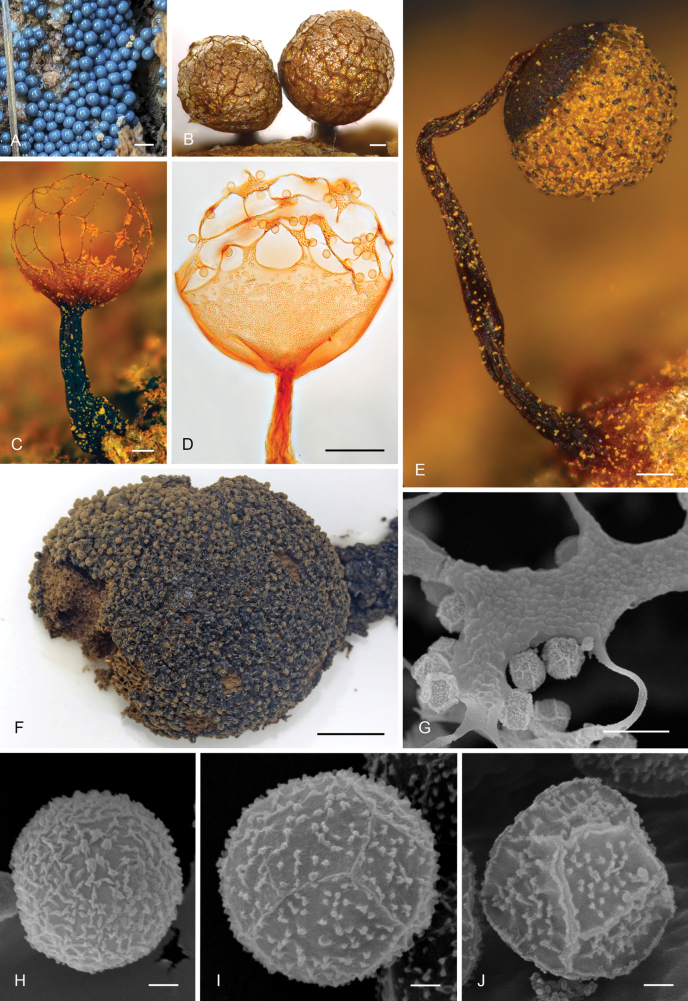
Morphological traits in Cribraria subg. Cribraria. A Bluish-grey sporophore initials of *C. argillacea* (G00586226); B–E sporocarpic sporophores of *C. argillacea* (B, G00586004, very shortly stalked), *C. rufa* (C, Koskinen JX.1648888#5), *C. minutissima* (D, Lado 27818, mounted in Hoyer’s medium), and C. cf. piriformis (E, G00586042); F nearly aethaliate sporophore of *C. tubulina* (G00586002); G node of *C. vulgaris* (MA-Fungi 61604) with encrusted dictydine granules; H–J spores under the scanning electron microscope of *Cribraria* sp. (H, Lloyd 1988), *C. minutissima* (I, Lado 27818) and *C. vulgaris* (J, MA-Fungi 61604). Scale bars: 1 mm (A); 0.1 mm (B, C, E); 50 µm (D); 1 cm (F); 10 µm (G); 1 µm (H–J).

The third lineage (Cribraria subg. Cribraria) received high support (SH-like aLRT = 81%, BS = 93%, TBE = 99%), except for the Bayesian analysis (PP = 0.91), and includes all the remaining species (Fig. 5) that usually have light grey to bluish-grey or dark lead grey emerging and fruiting plasmodia (Fig. 5A), but other colours may occur (e.g., green in *C. aurantiaca* Schrad., brick red in *C. minutissima* Schwein., blackish in *Lindbladia tubulina*), and sporophores with dictydine granules concentrated in the peridium (Fig. 5G) and sometimes in the stalk (some granules are occasionally present in the spore mass), at least in the analysed species. The sporophores in this group are usually of duller colours, frequently ochraceous, orangish or brownish (Fig. 5B–F), occasionally reddish or with subtle violaceous or purplish hues but not deeply violet or pink-purple. The sporophore morphologies are diverse, from long-stalked, isolated sporocarps (Fig. 5C–E), to nearly aethaliate sessile sporophores (Fig. 5F), including sporocarps that can be variously aggregated and with short stalks (Fig. 5B). The mature peridium remains as a basal calyculus (sometimes very reduced) and a net in the upper part of the sporotheca, with (Fig. 5E) or without (Fig. 5B–D) well-defined nodes that may be flat or thickened, occasionally some ribs may be present in the lower part, or it may exceptionally be unperforated (Fig. 5F). The spore ornamentation is variable, but the two most frequent types are irregularly and shortly ridged (Fig. 5H) and verrucose-spiny (Fig. 5I–J). In some cases, additional ridges to grooved long crests may be present, as in *C. rubiginosa* Fr. and *C. vulgaris* Schrad. (Fig. 5J).

**Figure 6.**
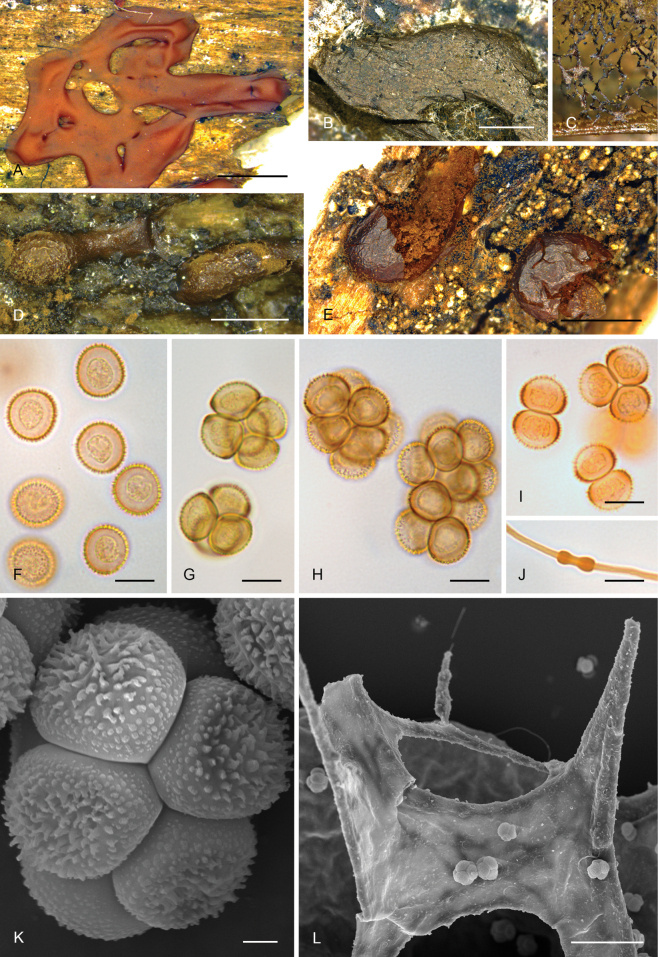
Morphological traits in the genus *Enteridium*. A Dried sclerotized plasmodium of *E. variabile* (≡ *Licea variabilis*) (G00586174) still showing a bright orange pigmentation; B–D mature sporophores of: *E. olivaceum* (B, C, Meyer 47160, image C showing a detail of the reticulate and membranous pseudocapillitium), *E. variabile* (D, G00586174), and *E. corticatum* (≡ *Dianema corticatum*) (E, Meyer 24378); F–I spores under the light microscope of *E. variabile* (F, G00586174), *E. olivaceum* (G, Meyer 47160), E. cf. liceoides (H, Woerly 3435a), and *E. corticatum* (I, Meyer 24378); J true capillitium of *E. corticatum* (Meyer 24378) with typical bead-like thickenings; K, L scanning electron microphotographs of *E. olivaceum* (MA-Fungi 57408) showing a spore cluster (K) and pseudocapillitial plates (L). Scale bars: 1 mm (A, B, D, E); 0.2 mm (C); 10 µm (F–J); 2 µm (K); 50 µm (L).

All samples of *Enteridium* are nested within the order *Trichiales* and within the family *Dianemataceae* T. Macbr., with high support (SH-like aLRT = 89%, BS = 75%, TBE = 97%, PP = 1). Two monophyletic groups are resolved in *Dianemataceae*, one of them comprising species in the genera *Prototrichia* Rostaf. and *Calomyxa* Nieuwl., as well as species of *Dianema* Rex with verrucose spores but excluding *D. corticatum* Lister, all of them with relatively abundant true capillitium. The sister group, recognized in this study as the genus *Enteridium* (Fig. 6, see discussion), received maximum support and comprises three main supported (SH-like aLRT = 95–100%, BS = 97–100%, TBE = 98–100%, PP = 1) clades: the first one, “*Dianema corticatum* clade”, with all samples of *D. corticatum* and one identified as *E. liceoides*; the second one, “*Licea variabilis* clade”, with all samples of *L. variabilis* Schrad.; and the third clade, “*Enteridium olivaceum* clade”, with all samples of *E. olivaceum* plus one sample identified as *E. liceoides* (Lister) G. Lister and another as *E. simulans* Rostaf. The studied samples have a reticulate and membranous pseudocapillitium (*E. olivaceum*, Fig. 6C, L), a true but not abundant filamentous capillitium (*D. corticatum*, Fig. 6J), or lack any capillitium or pseudocapillitium (*L. variabilis*). The spores may vary from isolated, rounded, and uniformly ornamented (e.g., *L. variabilis*, Fig. 6F) to clustered, slightly pyriform, and ornamented only in the exposed part of the cluster (e.g., *D. corticatum*, Fig. 6I; *E. olivaceum*, Fig. 6G, K).

## ﻿Discussion

### ﻿Morphological and molecular data in agreement: Enteridium belongs to the order Trichiales

As commented in the introduction, Leontyev et al. (2019) included the genus *Enteridium* (as *Licaethalium*) within the order *Cribrariales*, justifying their choice first by a previously published analysis of highly deviating nrSSU sequences ascribed to *Enteridium* (Leontyev et al. 2015), and second by the presence of three supposedly synapomorphic morphological characters of *Enteridium* and *Lindbladia*: a) the olive pigmentation of the spore mass, b) the black colour of immature fructifications, and c) the verrucose spores (i.e., the type of spore ornamentation). Our results disagree with these conclusions and place all studied samples of *Enteridium* within the order *Trichiales* (Fig. 1).

Regarding the DNA data, the highly deviating sequences of *Enteridium olivaceum* (as *Reticularia olivacea* [Ehrenb.] Fr.) from Leontyev et al. (2015) and the topology of the phylogeny presented in that publication are easily explained upon closer examination of the data generated for that study. A BLAST (“megablast”) search of the sequence KP941455 obtained from the specimen CWU 2312 shows a very high identity (99.59%) and good coverage (89%) to some species of the genus *Pinus*, such as *P. sylvestris*, *P. nigra*, *P. contorta*, etc. *Enteridium olivaceum* is frequently found on *Pinus* wood, and cross-contamination with *Pinus* material, such as pollen grains, is often observed in samples of *Myxomycetes* growing on that substrate. On the other hand, the sequence KP941454 from CWU 3075 is very similar to another sequence from *E. olivaceum* (OQ658907 from LE325311) generated as a barcode in Novozhilov et al. (2024), as well as to all our own nrSSU sequences of various specimens of *Enteridium olivaceum* s.l.; the closest BLAST results in this group of sequences are all with species of the order *Trichiales*.

**Figure 7.**
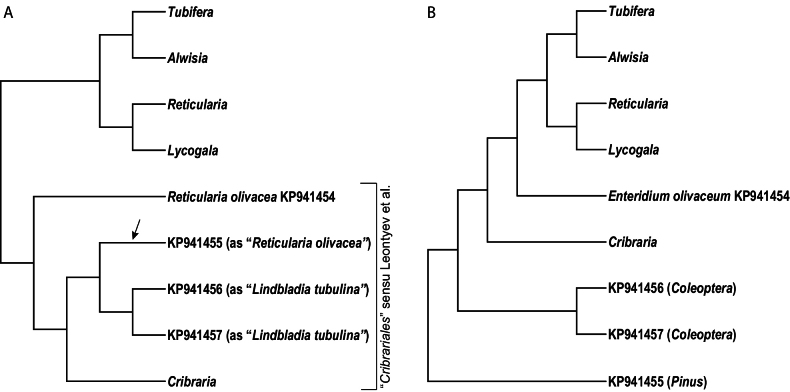
Simplified phylogenetic tree from Leontyev et al. (2015), in A As rooted and interpreted in that study and in B as interpreted by us according to the BLAST results and our data (see discussion). The arrow indicates the branch we used for re-rooting.

Interestingly, in the phylogeny proposed by Leontyev et al. (2015) (see a schema in Fig. 7A), the sequence KP941455 resembles more closely related to two sequences (KP941456 and KP941457) obtained from *Lindbladia tubulina* specimens (CWU MR 0119 and CWU 2311, respectively) than to the other sequence of *E. olivaceum* (KP941454, from CWU 3075). BLAST searches of the two supposedly *L. tubulina* sequences (KP941456 and KP941457) show a high identity (> 98%) and coverage (≥ 94%) to several sequences from beetle species of the families *Erotylidae* (*Pharaxonotha floridana*, *Pselaphacus nigropunctatus*), *Sphindidae* (*Protosphindus chilensis*), *Chrysomelidae* (*Aphthona* spp., *Phaedon tumidulus*, *Luperus sulphuripes*), etc. These sequences are, however, very different to those of *Lindbladia* used by Fiore-Donno et al. (2013) and those generated by us in the present study. Sugiura et al. (2019) reported the interactions between different species of beetles and *Myxomycetes*, the former possibly aiding at the spore dispersal of the latter. *Linbladia tubulina* was the sixth species of *Myxomycetes* with the highest number of observed interactions, especially with members of the family *Sphindidae*. Therefore, we consider that the sequences KP941455 from CWU 2312 (as *R. olivacea*) and the sequences KP941456 and KP941457 from CWU MR 0119 and CWU 2311 (as *L. tubulina*) most likely represent contaminations.

We found that the phylogenetic relationships presented in Leontyev et al. (2015) are misleading due to an inappropriate rooting (Fig. 7A). Following the BLAST results (Fig. 7B), if the phylogeny is rooted by using the most distant organism (a plant of probably the genus *Pinus*, sequence KP941455), then a clade with two animal sequences (*Coleoptera*, KP941456 and KP941457) appears sister to a main clade with only bright-spored *Myxomycetes* sequences. Within this *Myxomycetes* clade, three main lineages are identified, the first diverging one being the *Cribrariales* (with only *Cribraria*), and then another clade with *Reticulariales* (including *Tubifera* J.F. Gmel., *Alwisia* Berk. and Broome, *Reticularia*, and *Lycogala* Adans.) and *Trichiales* (with *Enteridium olivaceum*KP941454), exactly as expected and reported in other studies treating these taxonomic groups (e.g., Fiore-Donno et al. 2013), including ours (Fig. 1). *Enteridium* is, therefore, not nested in *Cribrariales*.

Concerning the morphology of *E. olivaceum*, both the colour of the spore mass and the spore ornamentation (Fig. 6K) agree perfectly with the ample variety of morphologies present in the order *Trichiales*. Indeed, the spore morphology (Fig. 6G, K) is much closer to that of some species of *Trichiales* (e.g., *Dianema corticatum*, Fig. 6I) than to any species of *Cribrariales*, within which clustered and unevenly ornamented spores are currently unknown (Figs 3–5). The spore ornamentation does justify the exclusion of *Enteridium* from the order *Reticulariales*, as Leontyev et al. (2015) stated. Regarding the colour of the immature fructifications, *E. olivaceum* is only dark-coloured when the sporophores are almost mature, while the plasmodium and very young fructifications are bright pinkish orange, pinkish red, or pink as repeatedly stated in the literature (see, e.g., Lister 1911; Martin 1949; Martin and Alexopoulos 1969; Nannenga-Bremekamp 1991), a colour unknown in the *Cribrariales* but present in many species of *Trichiales* (see, e.g., Martin and Alexopoulos 1969, see also Fig. 6A).

Therefore, both the molecular and the morphological data clearly place the genus *Enteridium* within the order *Trichiales*, specifically within the family *Dianemataceae*, of which *Enteridiaceae* M.L. Farr becomes a new heterotypic synonym.

### ﻿Why *Enteridium* and not *Licaethalium*?

The nomenclature of the generic names *Enteridium* and *Licaethalium* has been addressed by Leontyev and Ronikier (2024), but some details missing in that study are relevant for the nomenclatural stability of the involved names and deserve a closer analysis.

The generic name *Enteridium* was proposed by Ehrenberg (1819) to accommodate a single wood-inhabiting species forming aethalia, *E. olivaceum* Ehrenb. Ehrenberg’s description was rather detailed, indicating the presence of a membranous hypothallus, a peridium with bubbles or vesicles (resembling an intestine, hence the name), a netted or reticulate and membranous pseudocapillitium, and firmly conglobate spores in groups of 6–11, olivaceous in mass. The organism was found on rotten wood of *Alnus glutinosa* near Berlin (Ehrenberg 1819). The designation “*Enteridium*” had already been mentioned, but not validly published, in Ehrenberg’s dissertation (Ehrenberg 1818), just in a list, without description, diagnosis or any reference to a previously effectively published one but indicating that it was going to be published in “Jahrbücher de Gewächskunde von Sprengel Schrader und Link 1 Band 2 Heft”.

Later, Fries (1827) mentioned “*Reticularia olivacea*” in a list but neither a basionym nor even an indirect reference to Ehrenberg’s protologue was included, and Fries also failed to provide any diagnosis, description or further information, so “*Reticularia olivacea*” cannot be considered validly published there (Arts. 41.3 and 41.4 of the ICN do not apply). The same is true for the later edition entitled “Stirpium agri Femsionensis index”, dated in 1826 but surely published after 8 Dec 1827 (see Silva 1959; Stafleu and Cowan 1976), which Leontyev and Ronikier (2024) incorrectly considered the place of valid publication of *R. olivacea*. It is not until Fries (1829: 89) that *R. olivacea* was validly published. Fries (1829: 90) separately commented on Ehrenberg’s *Enteridium olivaceum* as: “*Enteridium olivaceum Ehrenb. […] habitu priori simillibum est, nullumque mihi dubium, quin status imperfectior in evolutione impeditus*”, which freely translates to “*Enteridium olivaceum* Ehrenb. […] is very similar to the former in habit, and I have no doubt that it is nothing more than an imperfect state hindered in development”. By this statement and by the adoption of the final epithet, we conclude that *R. olivacea* is a combination based on *E. olivaceum*, in agreement with previous studies (e.g., Martin and Alexopoulos 1969) and following the established custom.

On the other hand, Rostafiński (1873: 4) published *Licaethalium* as a new generic name, citing “Z.B. (zum Beispiel) *Reticularia olivacea* Fr., pag. 89”, an unambiguous reference to *Reticularia olivacea* (Ehrenb.) Fr., Syst. Mycol. 3: 89. 1829 (see above). Because *R. olivacea* is treated as a combination based on *E. olivaceum*, both names are necessarily homotypic, and *Licaethalium* becomes nomenclaturally superfluous and illegitimate due to the inclusion of the type of an earlier, legitimate name at the same rank (*Enteridium*), which name ought to have been adopted (Art. 52.1, ICN). The combination “*Licaethalium olivaceum*” was not validly published in 1873 because the epithet “*olivaceum*” was not definitely associated with the generic name “*Licaethalium*” (Art. 35.2, ICN). Soon after, Rostafiński (1875: 227) recognised that both generic names were synonyms and accepted *Enteridium* as the correct one, explaining that he was unaware of Ehrenberg’s name in his dissertation of 1873. Additionally, Rostafiński (1875) listed “*Licaethalium olivaceum* Rfski., Versuch., etc., p. 4” as if it had already been published in 1873, but this does also not constitute a valid publication of the name because it was merely cited as a synonym (Art. 36.1(b), ICN). Indeed, although Leontyev and Ronikier (2024) refer to “Rostafiński’s combination of *Licaethalium olivaceum*” in their study, we are not aware that “*Licaethalium olivaceum*” has ever been validly published. As a result, *Enteridium* and *E. olivaceum* are the correct names for Ehrenberg’s taxon for being the earliest legitimate ones in their respective ranks (Arts. 11.3 and 11.4, ICN).

### ﻿Updated taxonomy and nomenclature of *Enteridium*

As shown in our DNA-based phylogeny (Fig. 1), all specimens identified as *Enteridium olivaceum* belong to a fully supported clade that is closely related to two other morphologically similar species: *Licea variabilis* and *Dianema corticatum*. The similarity between *Enteridium* (including also *E. liceoides* and *E. simulans*) and these species has been noted several times by different authors (Lister 1911; Nannenga-Bremekamp 1974; Ing 1999), being unsurprising that they are evolutionarily related. Both *L. variabilis* and *D. corticatum* produce plasmodiocarps or irregular sporocarps (Fig. 6D, E) that may resemble those of *E. liceoides* or small sporophores of *E. olivaceum*. The three species *E. olivaceum*, *L. variabilis*, and *D. corticatum* form a well-supported clade that can be recognized as the genus *Enteridium* (see diagnosis in the taxonomic treatment). The two possible alternatives to this choice are (i) the recognition of a different genus for each of these three species or species complexes, an option that we consider as highly oversplitting, or (ii) *Enteridium* could encompass the whole family *Dianemataceae*, including *Calomyxa*, *Dianema* s.str., and *Prototrichia* (see also comments in García-Cunchillos et al. 2022), but this option is unsustained and discouraged until having a deeper knowledge on the diversity of these putative genera, as it would imply a non-negligible taxonomic lumping. *Enteridium* is nevertheless the oldest name and, as conservatively defined here, requires new combinations only for *L. variabilis* and *D. corticatum* (see below).

Two more species have been usually, but not always, accepted in *Enteridium* s.str.: *Enteridium simulans* and *E. liceoides*. The former was published by Rostafiński (1876) indicating, in comparison with *E. olivaceum*, that the spores were not clustered and that they were uniformly ornamented with small warts. One of the specimens included in our analyses (M. Meyer 29889 in herb. G) was identified as *E. simulans* because, indeed, the spores are mostly free when mounted, e.g., in Hoyer’s medium. However, the spores only have verrucae on one of their sides, and their shape is identical to those from typical specimens of *E. olivaceum*. The molecular data place this sample within the clade of *E. olivaceum* (Fig. 1), and it is possible that it represents a specimen of this species where the spore clusters are particularly fragile and dissociate easily, rather than truly being *E. simulans* in its original sense. This kind of easily dissociating spore clusters has been observed in other species of the family *Dianemataceae* (see *Dianema* sp. 1 in Ronikier et al. 2020). Martin and Alexopoulos (1969) stated that all intermediates between clustered and free spores exist and considered *E. simulans* as a synonym of *E. olivaceum*, while Nannenga-Bremekamp (1991) treated *E. simulans* as a variety of *E. olivaceum*. With the expressed doubts about the identification of the specimen of *E. simulans* included in our analyses, and waiting for further data, we provisionally accept this species based on the information from its protologue.

*Enteridium liceoides* was originally described as a variety of *E. olivaceum* by Lister (1896) and later risen to species level by her daughter Gulielma Lister (Lister 1919), being distinguished from *E. olivaceum* by producing isolated plasmodiocarps, simple or combined into a flat net, and by the very reduced pseudocapillitium. Two specimens identified as *E. liceoides* fall in two different groups in our phylogeny (Fig. 1), one is nested in the *E. olivaceum* clade, while another is closely related to *Dianema corticatum*, but neither molecularly nor morphologically identical to it (Fig. 6H, I). It may be possible that the morphology of *E. liceoides* corresponds to reduced forms lacking capillitium or pseudocapillitium, and the revision and detailed analyses of original material would be critical to assess its true identity. We provisionally accept it based on data from the literature and the distinctiveness of at least one of the analysed specimens. In any case, the species-level taxonomy of this group is out of the scope of the present study and needs to be investigated with a larger sampling of specimens (including type material) and DNA regions before reaching firm conclusions.

According to our morphological and molecular data, the following treatment of the genus *Enteridium* is proposed:

#### ﻿ Enteridium

Taxon classificationAnimaliaCribrarialesDianemataceae

Ehrenb., Jahrb. Gewächsk. 1(2): 55. 1819

622EFCC8-6B96-512B-A50E-782F40F9C184

12084

Fig. 6

≡ Licaethalium Rostaf., Vers. Syst. Mycetozoen: 4. 1873, nom. illeg. (Art. 52.1).

##### Type.

*Enteridium olivaceum* Ehrenb., Jahrb. Gewächsk. 1(2): 57. 1819.

##### Updated diagnosis.

Within the family *Dianemataceae*, *Enteridium* is characterized by the flat, usually effused sporophores with either a pseudocapillitium consisting of perforated plates or flat pillar-like structures, a true capillitium of sparse, simple threads, or lacking any capillitium or pseudocapillitium, and free or clustered spores, ornamented with warts or spines, these concentrated on the outer side of the cluster when the spores are not free.

##### ﻿Accepted species names

#### ﻿ Enteridium olivaceum

Taxon classificationAnimaliaCribrarialesDianemataceae

Ehrenb., Jahrb. Gewächsk. 1(2): 57. 1819

B5A79A2C-A8E0-591C-BD8B-A96D612126A9

148249

≡ Reticularia olivacea (Ehrenb.) Fr., Syst. Mycol. 3: 89. 1829.

#### ﻿ Enteridium simulans

Taxon classificationAnimaliaCribrarialesDianemataceae

Rostaf., Sluzowce monogr. suppl.: 30. 1876

905DB41A-DBB8-5096-97A1-AEBB440AAF33

222329

≡ Reticularia olivacea var. simulans (Rostaf.) Nann.-Bremek., Proc. Kon. Ned. Akad. Wetensch., C. 76(5): 486. 1973.
≡ Reticularia simulans (Rostaf.) D.W. Mitch., Syst. Geogr. Pl. 74: 261. 2004

#### ﻿ Enteridium liceoides

Taxon classificationAnimaliaCribrarialesDianemataceae

(Lister) G. Lister in Lister, Guide Brit. Mycetozoa, ed. 4: 48. 1919

DA41D9A0-5493-549B-BB69-4F9E1599F5FE

269378

≡ Enteridium olivaceum var. liceoides Lister, J. Bot. 34: 211. 1896 [basion.].
≡ Reticularia liceoides (Lister) Nann.-Bremek., Proc. Kon. Ned. Akad. Wetensch., C. 76(5): 485. 1973.

#### ﻿ Enteridium variabile

Taxon classificationAnimaliaCribrarialesDianemataceae

(Schrad.) J.C. Zamora, D. Rodrigues, García-Cunch. and Lado comb. nov.

A0227AFB-9AF6-5821-948D-83686D4A22B9

860087

≡ Licea variabilis Schrad., Nov. gen. pl. 18. 1797 [basion.].
≡ Tubulina variabilis (Schrad.) Poir., in Lamarck, Encycl. 8:131. 1808.

#### ﻿ Enteridium corticatum

Taxon classificationAnimaliaCribrarialesDianemataceae

(Lister) J.C. Zamora, D. Rodrigues, García-Cunch. and Lado comb. nov.

B89A15A4-8FD9-5BFA-BFB1-E2B84931B7A7

860088

≡ Dianema corticatum Lister, Monogr. Mycetozoa 1: 205. 1894 [basion.].

Finally, there are some currently accepted species that could not be analysed in our study, but which might as well be included in the genus *Enteridium*. Both *Enteridium aureum* (Nann.-Bremek.) M.L. Farr (≡ *Reticularia aurea* Nann.-Bremek.) and *E. rubiginosum* Gràcia, Illana and G. Moreno (≡ *Reticularia rubiginosa* [Gràcia, Illana and G. Moreno] Lado) share a similar spore ornamentation and are surely members of the order *Trichiales* (the type of *E. rubiginosum* was requested to AH herbarium, but it is apparently missing). Leontyev and Ronikier (2024) hypothesised that these two species may belong to the genus *Dictydiaethalium*. On the other hand, *Dianema repens* Lister was described indicating a close similarity to *D. corticatum* G. Lister and Cran but with slender plasmodiocarps, a thin membranous peridium, and coarse capillitium threads with membranous expansions (Lister 1925). In light of our results showing that *Enteridium* also belongs to *Trichiales* and is not necessarily restricted to species with clustered olivaceous spores and pseudocapillitium, we cannot discard the alternative hypothesis of any of the abovementioned species being included in *Enteridium*.

### ﻿Diagnosis of the order *Cribrariales*, taxonomy, and nomenclature

With the exclusion of *Enteridium* from the *Cribrariales*, the diagnosis of this order, in strict sense, returns to the broadly accepted conception of its single family *Cribrariaceae*, as defined and accepted in a plethora of publications (Lister 1925; Martin and Alexopoulos 1969; Nannenga-Bremekamp 1974, 1991, 2022; Lado and Pando 1997; Ing 1999; Poulain et al. 2011). *Cribrariales* is characterized by the presence of the so-called “plasmodic” or “dictydine” granules, which are globular structures, usually hollow, containing calcium (Schoknecht 1975) and possibly phosphorus and other elements (Hatano et al. 1996). These last authors traced the use of the terms “plasmodic granules” and “dictydine granules” to Lister (1894) and Martin (1949), respectively. However, we found that Rex (1891) already used “plasmodic granules” to refer to these structures, and their presence in the mature plasmodia (McManus 1963; Lado et al. 1999, Fig. 2 in our study) indicate that this terminology is probably correct, being employed in a few publications, even recently (e.g., Ramírez-Ortega et al. 2017, as plasmodial granules). On the other hand, the term “dictydine granules” was already used by Meylan (1908) as “grains de plasma ou dictydine” and has become common in recent publications (Lado et al. 1999; Estrada-Torres et al. 2001; Hooff 2009; Poulain et al. 2011; Leontyev et al. 2019), unambiguously and uniquely referring to these structures as found in *Cribrariales*; it is therefore the term used in the present study. Other terminologies, such as lime globules (Nannenga-Bremekamp 1991, 2022, not recommended by Hatano et al. 1996) and calcic granules (Lado and Pando 1997; Lado et al. 2019; Lado and Eliasson 2022), can be found in the literature.

The transformation of the whole protoplasm surrounded by the peridium into spores is another synapomorphy of all known species of the order *Cribrariales*, lacking the capillitium or pseudocapillitum that is usually present in the *Reticulariales* and *Trichiales* (occasionally reduced or secondarily lost in some species, see García-Cunchillos et al. 2022). The peridium, as already noted by de Bary (1866), also shows some peculiarities, as it is typically formed by a very thin and evanescent membrane with thickenings persisting in the form of nodes, ribs, threads, a calyculus, or a combination of those, rarely almost entirely persistent (*L. tubulina*).

Our analyses provide some insights into the phylogenetic relationships of different species of *Cribrariales*. As noted in the results, there are three well-defined lineages in *Cribrariales* (Fig. 1) that can be characterized by morphological traits. We have noted that the colour of the emerging plasmodium, the distribution of the dictydine granules in the mature sporophores, and the overall pigmentation of the fruiting bodies are useful taxonomic characters. The colour of the plasmodium has been used for taxonomic purposes in *Cribrariaceae* since a long time ago, notably to distinguish *C. aurantiaca* (with a striking green plasmodium) from others (Meylan 1913; Nannenga-Bremekamp 1971). However, caution is advised when interpreting data from the literature because some authors have considered a “plasmodial” state even when the sporophores are almost fully formed, but still soft. As a result, it is possible to find reports of “whitish” plasmodia for some species (e.g., *C. rufa* [Roth] Rostaf., see Lister 1911; Martin and Alexopoulos 1969; Nannenga-Bremekamp 1991) referring to the developmental stage where the spore mass cleared up after the dictydine granules had migrated to the peridium, as shown in our Fig. 2F for *C. violacea*. In fact, Martin and Alexopoulos (1969) precisely indicate this change of colour for *C. oregana* and *C. pachydictyon*. The colours reported in our study are either from our own observations of fresh material or from photographs showing the development of a specimen.

The earliest-diverging lineage of the family *Cribrariaceae* (Fig. 1) includes species that were segregated as the genera *Dictydium* and *Heterodictyon* in the past (Rostafiński 1873; Lister 1894; Martin and Alexopoulos 1969), the types of which are *D. umbilicatum* (= *C. cancellata*) and *H. mirabile* (≡ *C. mirabilis*), respectively. The fact that *C. mirabilis* is closely related to *C. cancellata* and should belong to the same group has been broadly accepted (Lister 1925; Nannenga-Bremekamp 1962; Martin and Alexopoulos 1969), and is fully supported by our results (Fig. 1). Therefore, *Heterodictyon* is simply considered as a later synonym of *Dictydium* because the alternative option would imply the recognition of a genus for almost every species. The distinction between *Dictydium* and *Cribraria* is more open to debate. *Dictydium* was usually kept as an independent genus by North American authors (Macbride 1899; Hagelstein 1944; Martin and Alexopoulos 1969; Keller and Braun 1999), and also in Europe (Lister 1925) until Nannenga-Bremekamp (1962) proposed to unite all species under *Cribraria*. We also consider that the best option with the current data is to keep *Cribraria* unsplit, thus retaining a genus that is easily and intuitively recognized, even in the field, by both specialists and amateurs. Within this lineage, *Cribraria purpurea* occupies a relatively isolated position. This distinctive species has very large dictydine granules (usually 3–4 µm in diam., sometimes larger) that are typically aggregated in packages in the spore mass (Fig. 3G), but rarely adhering to the spores themselves. Even with this unique characteristic, we think it is preferable to keep *C. purpurea* within the *Dictydium* group, considering that other species share characters with it; for example, *C. meylanii* also has a sporotheca with a well-developed calyculus and a fully reticulate peridium (Fig. 3C, F), pink to purplish pigments leaching in Hoyer’s medium are observable in specimens of *C. meylanii* and *C. cancellata* (Fig. 3F), and the early stages of development of *C. purpurea* and *C. cancellata* are very similar, with blackish emerging plasmodia (Fig. 3A) that sometimes stain the substrate of pink-purple.

The second lineage corresponds to the species with dark violet pigments. Previous studies have reported a dark violaceous plasmodium for *C. violacea* (Rex 1891; McManus 1963) and *C. zonatispora* (Lado et al. 1999), which agree with our own observations (Fig. 2). However, the developing plasmodium of the non-sequenced *C. tecta*, which probably belongs to this clade, is reported to be pale-coloured (Hooff 2009), but we lack data on the morphology of the concentrating plasmodium and sporophores initials. Other species that surely belong to this group, but from which we currently lack DNA data, are *C. fragilis* Lado and Estrada (Estrada et al. 2001), *C. bicolor* S.L. Stephenson, Novozh. and P. Wellman (Stephenson et al. 2018), and *C. spinispora* Lado and D. Wrigley (Lado et al. 2019). Interestingly, the unique spore ornamentation of *C. fragilis* (Fig. 4I) is somehow intermediate between those of *C. zonatispora* and *C. violacea*, adding to the large morphological variation displayed in this clade.

The third lineage contains the majority of studied specimens. The earliest-diverging species in this group is *C. minutissima* (Figs 1, 5D, I). To our knowledge, this is the only species of *Cribraria* with brick red emerging plasmodia (personal observations in the field), and the dictydine granules are frequently paler than in most other *Cribraria* species. Otherwise, *C. minutissima* is morphologically a typical member of C. subg. Cribraria that was even considered conspecific with *C. confusa* Nann.-Bremek. and Y. Yamam. until Nannenga-Bremekamp and Yamamoto (1983) definitely separated them. For this reason, some reports from the literature indicating a dark bluish plasmodium for this species (Lister 1925; Martin and Alexopoulos 1969) should be taken with caution, as those observations may refer to *C. confusa*. Our results confirm that *Lindbladia* is deeply nested within this third lineage, as shown by Fiore-Donno et al. (2013). The morphological similarity between *Lindbladia* (Fig. 5F) and one species of *Cribraria*, *C. argillacea* (Pers. ex J.F. Gmel.) Pers. (Fig. 5A, B), has been pointed out in several publications, even noting that intermediate forms occur (Rex 1892; Lister 1894; Macbride 1922; Nannenga-Bremekamp 1974, 2022). Because *Lindbladia tubulina*, the type of the genus *Lindbladia*, belongs to the same group of *C. rufescens* (= *C. rufa*, Fig. 5C), the type of *Cribraria*, and it is very closely related to other species of typical *Cribraria*, such as *C. argillacea*, *Lindbladia* cannot be kept as an independent genus without describing a large number of putative new genera that would be difficult to diagnose. This option is strongly discouraged because it would imply an excessive and unnecessary over splitting. We then accept *L. tubulina* as a species of *Cribraria*, requiring the following combination: *Cribraria tubulina* (Fr.) J.C. Zamora, D. Rodrigues, García-Cunch. and Lado, comb. nov. (≡ *Lindbladia tubulina* Fr., Summa veg. Scand.: 449. 1849 [basion.]; MycoBank MB 860089). This decision is taken in parallel with other studies showing the occurrence of aethalic morphologies within genera mainly composed of sporocarpic species, such as the inclusion of the former genus *Mucilago* within *Didymium* in the order *Physarales* (García-Martín et al. 2023).

The three lineages indicated above are morphologically distinguishable and well-supported by our molecular analyses, and their recognition as infrageneric taxa may be useful in a genus where species are often very difficult to distinguish. As a result, we decide to treat them as the following three subgenera. We cite a non-exhaustive list of included species to help define our current concept of these three groups and those included in our molecular analyses (albeit sometimes with an uncertain identification or treated in broad sense) are in bold. Some species, with an uncertain placement, are purposely omitted.

#### ﻿ Cribraria

Taxon classificationAnimaliaCribrarialesCribrariales

Pers., Neues Mag. Bot. 1: 91. 1794 nom. cons.

3750D614-1B40-50C7-B950-C84420931321

##### Note.

See Gams 2005; Lado et al. 2005.

#### ﻿ Cribraria

Taxon classificationAnimaliaCribrarialesCribrariales

Pers., Neues Mag. Bot. 1: 91. 1794 subg. Cribraria

4DA670E0-0A6F-5614-BB1D-9ADC7F8096F3

12058

Fig. 5

= Cribraria subg. Schraderella Rostaf., Sluzowce monogr.: 232. 1875 ― MycoBank MB 860090. Type [designated here, MBT 10027663]: Cribraria rufa (Roth) Rostaf.
= Lindbladia Fr., Summa veg. Scand. 449. 1849. Type: L. tubulina Fr., Art. 40.2, ICN.
– Cribraria subg. Eucribraria Rostaf., nom. inval., Art. 21.3, ICN.

##### Type.

*Cribraria rufescens* Pers. (= *C. rufa* [Roth] Rostaf.), designated by Martin (1949: 26).

##### Diagnosis.

Emerging plasmodium usually greyish or bluish grey (light grey to dark lead grey), occasionally of other colours (e.g., green, brick red, blackish). Mature sporophores aethaliate, pseudoaethaliate or sporocarpic, sessile or stalked, yellowish, ochre, copperish, orange, brownish red, or light to dark brown, with dictydine granules concentrated on the peridium, sometimes also present in the stalk.

##### Species included.

*C. angulospora* C.H. Liu and J.H. Chang, *C. argillacea* (Pers. ex J.F. Gmel.) Pers., *C. aurantiaca* Schrad., *C. confusa* Nann.-Bremek and Y. Yamam., *C. cribrarioides* (Emoto) Hatano, *C. dictyospora* G.W. Martin and Lovejoy, *C. filiformis* Nowotny and H. Neubert, *C. gothica* Sadykov, *C. intricata* Schrad., *C. languescens* Rex, *C. macrocarpa* Schrad., *C. macrostipitata* H. Neubert and Nann.-Bremek., *C. martinii* Nann.-Bremek., *C. microcarpa* (Schrad.) Pers., *C. minutissima* Schwein., *C. oregana* H.C. Gilbert s.l. (including *C. montana* Nann.-Bremek.), *C. pachydictyon* Nann.-Bremek., *C. paucidictyon* Yu Li, *C. persoonii* Nann.-Bremek., *C. pertenuis* Flatau and Schirmer, *C. piriformis* Schrad., *C. rubiginosa* Fr., *C. rufa* (Roth) Rostaf., *C. splendens* (Schrad.) Pers., *C. stellifera* Nowotny and H. Neubert, *C. tenella* Schrad., *C. tubulina* (Fr.) J.C. Zamora, D. Rodrigues, García-Cunch. and Lado, *C. vulgaris* Schrad.

#### ﻿ Cribraria subg. Dictydium

Taxon classificationAnimaliaCribrarialesCribrariales

(Schrad.) Y. Yamam., The Myxomycete biota of Japan: 94. 1998

582631C9-DEEC-5449-89FB-A9B60A068868

859338

Fig. 3

≡ Dictydium Schrad., Nov. gen. pl.: 11. 1797 [basion.].
= Heterodictyon Rostaf., Vers. Syst. Mycetozoen: 5. 1873. Type: H. mirabile Rostaf., Art. 40.2, ICN.

##### Type.

*Dictydium umbilicatum* Schrad. (= *Cribraria cancellata* [Batsch] Nann.-Bremek.), designated by Martin (1949: 32).

##### Diagnosis.

Emerging plasmodium very dark brown to blackish, sometimes with purplish hues. Mature sporophores always sporocarpic, stalked, brownish, maroon to pink-purplish, with dictydine granules abundantly present both in the peridium and in the spore mass (attached to the spores or not), sometimes also present in the stalk.

##### Species included.

*C. cancellata* (Batsch) Nann.-Bremek., *C. mirabilis* (Rostaf.) Massee, *C. meylanii* Brândza, *C. purpurea* Schrad., *C. rutila* (G. Lister) Nann.-Bremek.

##### Nomenclatural notes.

We consider the name at subgeneric rank as accepted by Yamamoto (1998), where all requirements for its valid publication as a combination are fulfilled. Yamamoto (op. cit.) ascribed the authorship to “(Schrad.) Pers.” noting that in Persoon (1801) this group was referred to as “*Dictydia*”. Persoon (op. cit.: 189) indeed mentioned “*Dictydia* Schrader” referring to the group of species that were treated by Schrader (1797) as *Dictydium*, but there is no indication that Persoon accepted an infrageneric name for this species group and, in any case, such a name would be unranked and not compete for purposes of priority (Art. 37.3, ICN).

#### ﻿ Cribraria subg. Ionokylix

Taxon classificationAnimaliaCribrarialesCribrariales

J.C. Zamora, D. Rodrigues, García-Cunch. & Lado subg. nov.

B035E3ED-7DFD-5B4A-9E16-9BD8086F276E

860091

Fig. 4

##### Etymology.

Neuter compound noun from the Greek prefix “ῐ̓́ονo-”, referring to violet, and the word “κύλιξ”, an ancient Greek pottery used to drink wine, due to the colour of the cup-like peridium.

##### Type.

*Cribraria violacea* Rex.

##### Diagnosis.

Emerging plasmodium usually violaceous. Mature sporophores always sporocarpic, stalked and violet to dark violet, with dictydine granules concentrated on the peridium, sometimes also present in the stalk.

Species included: *C. bicolor* S.L. Stephenson, Novozh. and P. Wellman, *C. fragilis* Lado and Estrada, C. aff. lepida Meyl., *C. spinispora* Lado and D. Wrigley, *C. tecta* Hooff, *C. violacea* Rex, *C. zonatispora* Lado, Mosquera and Beltrán-Tej.

## Supplementary Material

XML Treatment for Enteridium

XML Treatment for Enteridium olivaceum

XML Treatment for Enteridium simulans

XML Treatment for Enteridium liceoides

XML Treatment for Enteridium variabile

XML Treatment for Enteridium corticatum

XML Treatment for Cribraria

XML Treatment for Cribraria

XML Treatment for Cribraria subg. Dictydium

XML Treatment for Cribraria subg. Ionokylix

## ﻿Additional information

### Use of AI

No use of AI was reported.

### Adherence to national and international regulations

All the fungal strains used in this study have been legally obtained, respecting the Convention on Biological Diversity (Rio Convention).

### Author ORCIDs

Juan Carlos Zamora https://orcid.org/0000-0002-9243-2999

Daniel Rodrigues https://orcid.org/0000-0001-8399-1222

Iván García-Cunchillos https://orcid.org/0000-0002-5830-7529

Carlos Lado https://orcid.org/0000-0002-6135-2873

### Data availability

All of the data that support the findings of this study are available in the main text or Supplementary Information.

## Supplementary materials

Supplementary material 1 Final alignment Data type nex Explanation note Alignment of mtSSU and nrSSU of bright-spored *Myxomycetes*, with *Physarales* as outgroup. https://binary.pensoft.net/file/1428817This dataset is made available under the Open Database License (http://opendatacommons.org/licenses/odbl/1.0/). The Open Database License (ODbL) is a license agreement intended to allow users to freely share, modify, and use this Dataset while maintaining this same freedom for others, provided that the original source and author(s) are credited.Author: Juan Carlos Zamora
